# Qualitative Evaluation of the Project P.A.T.H.S. Based on the Perceptions of the Program Participants

**DOI:** 10.1100/tsw.2006.354

**Published:** 2006-11-16

**Authors:** Daniel T.L. Shek, Tak Yan Lee, Andrew Siu, Ching Man Lam

**Affiliations:** ^1^ Quality of Life Centre, Hong Kong Institute of Asia-Pacific Studies, The Chinese University of Hong Kong, China; ^2^ Department of Applied Social Studies, City University of Hong Kong, China; ^3^ Department of Rehabilitation Sciences, The Hong Kong Polytechnic University, China; ^4^ Social Welfare Practice and Research Centre, The Chinese University of Hong Kong, Hong Kong, China

**Keywords:** positive youth development, Hong Kong, qualitative evaluation, focus group

## Abstract

Qualitative evaluation was carried out to understand the perceptions of the students participating in the Tier 1 Program of the P.A.T.H.S. Project. Five focus groups based on 43 students recruited from four schools were conducted to generate qualitative data to evaluate the program. With specific focus on how the informants described the program, results showed that the descriptors used were mainly positive in nature. When the informants were invited to name three metaphors that could stand for the program, the related metaphors were basically positive in nature. Finally, the program participants perceived many beneficial effects of the program in different psychosocial domains. Intra- and inter-rater reliability analyses revealed that the coding of the positive or negative nature of the responses was reliable. The present study provides qualitative support for the effectiveness of the Tier 1 Program of the Project P.A.T.H.S. in promoting holistic development in Chinese adolescents in Hong Kong.

## INTRODUCTION

According to Patton[1], there are two main approaches in the field of evaluation. On the one hand, the quantitative/experimental paradigm has the following characteristics: quantitative data (use of numbers and statistics), experimental designs, treatment and control groups, deductive hypothesis testing, objective perspective, evaluator aloof from the program, independent and dependent variables, linear and sequential modeling, pre-post focus on change, probabilistic and random sampling, standardized and uniform procedures, fixed and controlled designs, statistical analysis, as well as generalizations. On the other hand, the following attributes are intrinsic to the qualitative/naturalistic paradigm: qualitative data (e.g., narratives), naturalistic inquiry, case studies, inductive analysis, subjective perspective, evaluator close to the program, holistic contextual portrayal, systems perspective focused on interdependencies, dynamic and ongoing view of change, purposeful sampling of relevant cases, focus on uniqueness and diversity, emergent and flexible designs, thematic content analysis, and extrapolations. An examination of the field of prevention shows that although there is still a strong preference for the use of the quantitative/experimental approach to evaluate the effectiveness of prevention programs for adolescents, the number of qualitative evaluation studies in this area is increasing[2].

The Project P.A.T.H.S. (**P**ositive **A**dolescent **T**raining through **H**olistic **S**ocial Programmes) is a youth enhancement program that attempts to promote holistic youth development in Hong Kong[3]. There are two tiers of programs (Tier 1 and Tier 2) in this project. The Tier 1 Program is a universal positive youth development program based on 15 positive youth development constructs[4] in which students in Secondary 1 to 3 will take part. Obviously, to enable researchers to claim that the Tier 1 Program of the project is effective, research evidence is needed. Adopting the one-group pre- and post-test design, Shek[5] reported that there were positive changes in the students who joined the Tier 1 Program. The positive changes included enhancement in psychosocial competencies (social, emotional, cognitive, behavioral, and moral), resilience, self-determination, self-efficacy, beliefs in the future, and clear and positive identity. While the objective outcome evaluation findings are encouraging, it should be noted that there are limitations of the conclusions that can be derived from such a pre-experimental design. As such, it is argued that evaluation data based on qualitative methodology should also be conducted to examine the participants' perceptions of the program as well as the perceived program effects.

In their review of the common problems intrinsic to qualitative evaluation studies in the social work literature, Shek et al.[2] suggested that 12 principles should be maintained in a qualitative evaluation study. These include: explicit statement of the philosophical base of the study (Principle 1); justifications for the number and nature of the participants of the study (Principle 2); detailed description of the data collection procedures (Principle 3); discussion of the biases and preoccupations of the researchers (Principle 4); description of the steps taken to guard against biases or arguments that biases should and/or could not be eliminated (Principle 5); inclusion of measures of reliability, such as inter- and intra-rater reliability (Principle 6); inclusion of measures of triangulation in terms of researchers and data types (Principle 7); inclusion of peer- and member-checking procedures (Principle 8); consciousness of the importance and development of audit trails (Principle 9); consideration of alternative explanations for the observed findings (Principle 10); inclusion of explanations for negative evidence (Principle 11); and clear statement of the limitations of the study (Principle 12). In this qualitative evaluation study, the above principles were upheld as far as possible.

The purpose of this paper is to describe some of the qualitative findings based on focus group interviews with students participating in the Tier 1 Program. Although there are many strands of qualitative research[6], the most commonly used approach in qualitative research is the general qualitative approach where the general strategies of qualitative research are employed (e.g., collection of qualitative data, respecting the views of the informants, data analysis without a preset coding scheme), but a specific qualitative approach is not adhered to[7]. The exposition of the nature of this qualitative study is consistent with the view of Shek et al.[2] that there should be an explicit statement of the philosophical base of the study (Principle 1).

## METHODS

### Participants

Among the 52 schools joining the Experimental Implementation Phase, there were 29 schools adopting the full program (i.e., 20h program involving 40 units) and 23 schools adopted the 10h core program only. In the sampling process, eight randomly selected schools joining the full program and two randomly selected schools joining the core program were invited to participate in the focus group interviews. As the time for conducting the interviews was near the end of the term, three schools joining the full program and one school joining the core program declined our invitation to participate. As a result, five schools joining the full program (with one school expressing having difficulty to arrange group interviews with students) and one school joining the core program joined the focus group interviews (i.e., a total of six schools). For one school, the research team discovered at the time of the interview that untrained teachers had implemented the program, although the school had sent a sufficient number of teachers to join the training workshops held in the fall of 2005. As this practice seriously violated the program requirement and it seriously undermined the implementation quality of the program, the interview data based on this school were discarded. For another school, because space was enough to form two groups, two separate focus groups were conducted. In short, five focus groups based on students recruited from four schools (three schools joining the full program and one school joining the core program) participated in the focus group interviews.

For the consenting schools, the workers concerned randomly selected informants from the participating students to join the focus groups. As a result, 43 students participated in the focus group interviews, with 10 students (one focus group) from School A, 12 students (one focus group) from School B, 11 students (two focus groups) from School C, and 10 students (one focus group) from School D.

As the number of schools randomly sampled was roughly one-tenth of the participating schools, the number can be regarded as acceptable. Furthermore, the strategy of randomly selecting the schools, which is not commonly done in qualitative evaluation studies, can be regarded as an additional strength of the study. Finally, random selection of students from the students joining the Tier 1 Program can help to enhance the generalizability of the findings. The number of students joining the focus group discussion can also be regarded as acceptable. These arguments can satisfy Principle 2 (i.e., justifications for the number and nature of the participants of the study) proposed by Shek et al.[2].

## Procedures

The researchers and the research assistants individually or jointly conducted the focus group interviews. During the interviews, the participants were encouraged to verbalize their views and perceptions of the program. With respect to Principle 3 (i.e., detailed description of the data collection procedures) suggested by Shek et al.[2], the broad interview guide of the focus group interviews conducted is presented in Table 1. The interview questions were designed with reference to the CIPP model[8] and previous research[9]. In the interviews, the facilitators were conscious of the importance of adopting an open attitude to accommodate both positive and negative experiences expressed by the program participants. As the research assistants and researchers conducting the interviews either had training in social group work and/or substantial group work experience, they were conscious of the importance of encouraging the informants to express views of different nature, including both positive and negative views. After obtaining the consent of the participants, the focus group interviews were audio taped.

## Data Analysis

The interviews were tape recorded, and the content of the interviews was fully transcribed by student helpers and checked for accuracy by two research assistants. To enhance triangulation in the coding process, two research assistants and the first author were involved in the data analyses of the narratives. Our unit of analysis was a meaningful unit instead of a statement. For example, the statement that a program was "meaningful and amusing" would be broken down to two meaningful units or attributes, namely, "meaningful" and "amusing".

The present coding system was developed after much consideration of the raw data and several preliminary analyses. After initial coding, the positive or negative nature of the codes was determined, with four possibilities (positive code, negative code, neutral code, and undecided code). To enhance the reliability of the coding of the positive or negative nature of the raw codes, intra- and inter-rater reliability were carried out. Furthermore, descriptions with the same meaning (e.g., "good quality" and "high quality") were grouped into the same attribute category. Because of space limitation, qualitative findings on three areas are presented in this paper: (1) descriptors that were used by the informant to describe the program, (2) metaphors (i.e., incidents, objects, or feelings) that were used by the informants to stand for the program, and (3) participants' perceptions of the benefits of the program on themselves.

**TABLE 1**
**Interview Guide for the Focus Group Interviews Involving the Program Participants**

**A. Process Evaluation:**

**1. General Impression of the Program**
- What is your overall impression of the program? What are your feelings?
- Overall, did you enjoy participating in the program?
- With reference to the program, what has given you a lasting impression?
- Do you have any unforgettable experiences concerning your participation in this program?

**2. Comments on the Program Content**
- Were there any activities that most effectively aroused your interest to participate in them?
- Regarding the program, what are the things you like? What are the things you dislike?
- What are your views on the different units and content of the program?
- Which units do you like the most? Why?

**3. Comments on the Program Implementation**
- What are your thoughts on the degree or extent of participation of the entire class (i.e., all the students)?
- How do you feel about the atmosphere and discipline of the class when the program was implemented?
- What are the responses of the participating students regarding the program?

**4. Comments on the Instructors**
- What are your views on the instructors who conducted the program?
- Regarding the interactions between the instructors and students, what are your thoughts and feelings?

**B. Product Evaluation:**

**1. Evaluation of the General Effectiveness of the Program**
- Do you feel that the program is beneficial to the development of adolescents?
- Do you think that the program has helped your development?
- After participating in the program, do you have any changes? If yes, please specify. (free elicitation)
- What have you gained in this program? (free elicitation)
- If you feel that you have changed, what do you think are the factors that have promoted such changes?
- If you have not noticed any changes in yourself, what do you think are the reasons?

**2. Evaluation of the Specific Effectiveness of the Program**
- Do you think that your participation in the program has affected your school work and grades? Please elaborate your answers.
- Do you think the program can promote your self-confidence or ability to face the future?
- Do you think the program can enhance your abilities in different areas in your life?

Optional Questions
- Do you think the program can promote your spiritual life?
- Do you think the program can promote your bonding with family, teachers, and friends?
- Do you think the program can cultivate your compassion and care for others?
- Do you think the program can promote your participation in and care for the society?
- Do you think the program can promote your sense of responsibility to the society, family, school, and peers?

**3. Other Comments**
- If you are invited to use three descriptors to describe the program, what three descriptors will you use to describe the program?
- If you are invited to use one incident, object, or feeling (e.g., indigestion, enjoyment, etc.) to describe the program, what metaphor will you use to stand for the program?

### Ideological Biases and Preoccupations as Well as Strategies to Deal with Them

Shek et al.[2] argued for the importance of discussing the ideological biases and preoccupations of the researchers in a qualitative evaluation report (Principle 4). As program developers, the authors might have the preoccupation that the implemented program was good and it was beneficial to the students. In addition, the researchers might have the tendency to look at positive evidence rather than negative evidence. Thus, it is important to discuss how such biases were addressed in this study[2].

Several safeguards against the subtle influence of such ideological biases and preoccupations were included in the process of the study (Principle 5). First, the researchers were conscious of the existence of ideological preoccupations (e.g., positive youth development programs are beneficial to adolescents), and data collection and data analyses procedures were carried out in a disciplined manner. Second, although the analyses and interpretations were mainly carried out by the first author with the assistance of the two research assistants, inter- and intra-rater reliability checks on the coding were carried out (Principle 6). Third, multiple researchers and research assistants were involved in the data collection and analysis processes (Principle 7). Fourth, the first author was conscious of the importance and development of audit trails (Principle 9). The tapes, transcriptions, and steps involved in the development of coding system and interpretations were properly documented and systematically organized.

## RESULTS

For the descriptors used by the informants to describe the program, there were 71 raw descriptors and they could be further categorized into 22 categories (Table 2). Among these descriptors, 54 (76.1%) of them were coded as positive descriptors. In order to examine the reliability of the coding, the research assistants recoded 20 randomly selected raw descriptors (without knowing the original codes given) at the end of the scoring process. Intra-rater agreement percentages calculated from these descriptors were 100 and 100% for the two research assistants, respectively. Finally, these 20 randomly selected descriptors were coded by two colleagues with Ph.D. degrees without knowing the original codes given. Finding showed that the coded responses corresponded to those of the first author (95 and 85%, respectively).

For the metaphors that were used by the informants that could stand for the program, there were 35 raw "objects" involving 40 related attributes (Table 3). The findings showed that 27 metaphors (77.1%) can be regarded as positive metaphors and 33 attributes (82.5%) can be regarded as positive attributes. In order to examine the reliability of the coding, the research assistants recoded 20 randomly selected metaphors without knowing the original codes given at the end of the scoring process. Intra-rater agreement percentages calculated from these metaphors were 95 and 95% for the two research assistants, respectively. Finally, the metaphors were coded by two doctoral level colleagues, with high inter-rater agreement with the first author (90 and 85%, respectively).

Regarding the perceived benefits of the program on the program participants, 129 responses were recorded involving 40 attribute categories (Table 4). The findings showed that 124 responses (96.1%) were coded as positive responses. In order to examine the reliability of the coding, the research assistants recoded 20 randomly selected responses without knowing the original codes given at the end of the scoring process. Intra-rater agreement percentages calculated from these responses were 100 and 100%, respectively. Finally, the raw benefit categories were coded by two doctoral level colleagues without knowing the original codes given. Results showed that inter-rater agreement percentages between these raters and the first author were 95 and 100%, respectively.

## DISCUSSION

This paper attempts to examine the perceptions of the program participants regarding the qualities and effectiveness of the Tier 1 Program of the P.A.T.H.S. Project. Two major conclusions can be drawn from

**TABLE 2**
**Categorization of the Descriptors Used by the Participants to Describe the Program**

| Descriptors | Positive or Negative Nature of the Descriptor | | | | |
|---|---|---|---|---|---|
| | Positive | Neutral | Negative | Undecided | Total |
| Low cost | | | | 1 | 1 |
| Boring | | | 10 | | 10 |
| Senseless | | | 3 | | 3 |
| Repetitive | | | 1 | | 1 |
| Killing time | | | 1 | | 1 |
| Fair | | 1 | | | 1 |
| Fun, amusing | 10 | | | | 10 |
| Interesting | 7 | | | | 7 |
| Good, excellent | 6 | | | | 6 |
| Lively, exciting, not dull | 5 | | | | 5 |
| Meaningful | 4 | | | | 4 |
| Novel | 3 | | | | 3 |
| Relaxed | 3 | | | | 3 |
| Comfortable, enjoyable | 3 | | | | 3 |
| Happy | 3 | | | | 3 |
| Rich content | 2 | | | | 2 |
| Comprehensible | 2 | | | | 2 |
| Applicable, close to real life | 2 | | | | 2 |
| Useful | 1 | | | | 1 |
| Professional | 1 | | | | 1 |
| Better than other lessons | 1 | | | | 1 |
| Efficient | 1 | | | | 1 |
| **Total Count (Percentage):** | **54 (76.1%)** | **1 (1.4%)** | **15 (21.1%)** | **1 (1.4%)** | **71 (100%)** |

the qualitative evaluation findings obtained in this study. First, the program was basically perceived in a favorable light from the perspective of the program participants (Tables 2 and 3). Although some students perceived the program to be boring, this was not the dominant view, and some participants perceived the program to be amusing and exciting. Such negative findings are consistent with the observation of Shek[5] that roughly 15% of the participants did not perceive the program to be effective.

Second, the findings in Table 4 strongly suggest that most of the participants perceived the program to be beneficial to them, with 96.1% of the responses coded as positive responses. Generally speaking, benefits in the domains of bonding (e.g., improved communication and relationship with family), resilience (e.g., enhanced stress management), social competence (e.g., improved communication skills and interpersonal relationship), emotional competence (e.g., enhanced ability in handling emotions), cognitive competence (e.g., enhanced critical thinking), behavioral competence (e.g., acquisition of refusal skills), moral competence (e.g., increased awareness of public morals), self-efficacy (e.g., enhanced self-efficacy), self-determination (e.g., enhanced self-confidence), beliefs in the future (e.g., helpful for future career), spirituality (e.g., helpful in understanding purpose of life), clear and positive identity (e.g., enhanced self-understanding), prosocial norms (e.g., enhanced respect for others) and prosocial involvement (e.g., engagement in voluntary work) were observed. These observations are consistent with the objective outcome evaluation findings of Shek[5] that the students changed in the positive direction in various outcome indicators. With reference to the principle of triangulation, the

**TABLE 3**
**Categorization of the Metaphors (Incidents, Objects, Feelings, etc.) Used by the Participants**

| Metaphors | Positive or Negative Nature of the Metaphor | | | | Number of Codes Derived from the Metaphor and Its Nature | | | |
|---|---|---|---|---|---|---|---|---|
| | Positive | Neutral | Negative | Total | Positive | Neutral | Negative | Total |
| Chalk | | | 1 | 1 | 0 | 0 | 1 | 1 |
| Soft drink | | | 1 | 1 | 0 | 0 | 1 | 1 |
| Eating | | | 1 | 1 | 0 | 0 | 1 | 1 |
| Boredom | | | 1 | 1 | 0 | 0 | 0 | 0 |
| Lack of motivation to learn | | | 1 | 1 | 0 | 0 | 0 | 0 |
| Sermon | | 1 | | 1 | 0 | 0 | 0 | 0 |
| Mother | | 1 | | 1 | 0 | 1 | 0 | 1 |
| Sweet and sour rib | | 1 | | 1 | 0 | 1 | 0 | 1 |
| Treasure of knowledge | 1 | | | 1 | 1 | 0 | 0 | 1 |
| Mirror | 1 | | | 1 | 1 | 0 | 0 | 1 |
| Plastic clay | 1 | | | 1 | 1 | 0 | 0 | 1 |
| Toured guide | 1 | | | 1 | 1 | 0 | 0 | 1 |
| Orange | 1 | | | 1 | 1 | 0 | 0 | 1 |
| Vacuum cleaner | 1 | | | 1 | 1 | 0 | 0 | 1 |
| Air purifier | 1 | | | 1 | 2 | 0 | 0 | 2 |
| Computer | 1 | | | 1 | 1 | 0 | 0 | 1 |
| Library | 1 | | | 1 | 3 | 0 | 0 | 3 |
| Play Station 2 | 1 | | | 1 | 1 | 0 | 0 | 1 |
| Teacher | 1 | | | 1 | 2 | 0 | 0 | 2 |
| Ice cream | 1 | | | 1 | 1 | 0 | 0 | 1 |
| Pen | 1 | | | 1 | 1 | 0 | 0 | 1 |
| Trendy | 1 | | | 1 | 1 | 0 | 0 | 1 |
| Cancer | 1 | | | 1 | 2 | 0 | 0 | 2 |
| Amusement | 1 | | | 1 | 0 | 0 | 0 | 0 |
| Rejuvenation | 1 | | | 1 | 0 | 0 | 0 | 0 |
| Mango juice | 1 | | | 1 | 1 | 0 | 0 | 1 |
| Drinking tea | 1 | | | 1 | 1 | 0 | 0 | 1 |
| Solid | 1 | | | 1 | 0 | 0 | 0 | 0 |
| Colorful and rich | 1 | | | 1 | 0 | 0 | 0 | 0 |
| A treasure box | 1 | | | 1 | 2 | 0 | 0 | 2 |
| Enlightening teacher | 1 | | | 1 | 0 | 0 | 0 | 0 |
| A living dictionary for life | 1 | | | 1 | 5 | 1 | 0 | 6 |
| Chewing gum | 1 | | | 1 | 1 | 0 | 1 | 2 |
| Music | 1 | | | 1 | 2 | 0 | 0 | 2 |
| A glass of clear water | 1 | | | 1 | 1 | 0 | 0 | 1 |
| **Total Count (Percentage):** | **27 (77.1%)** | **3 (8.6%)** | **5 (14.3%)** | **35 (100%)** | **33 (82.5%)** | **3 (7.5%)** | **4 (10%)** | **40 (100%)** |

present study and the previous findings suggest that based on quantitative and qualitative evaluation findings, evidence on the positive effect of the Tier 1 Program on holistic youth development among the program participants is present.

**TABLE 4**
**Categorization of Responses on the Perceived Benefits of and Things Learned in the Program**

| Benefits | Nature of the Response | | | | |
|---|---|---|---|---|---|
| | Positive | Neutral | Negative | Undecided | Total |
| Cannot learn anything | | | 2 | | ***2*** |
| Unhelpful | | | 2 | | ***2*** |
| Not much change | | 1 | | | ***1*** |
| **Character:** | | | | | |
| Enhanced self-understanding | 4 | | | | 4 |
| Positive personality change | 4 | | | | 4 |
| Enhanced self-reflection | 1 | | | | 1 |
| Enhanced self-discipline | 1 | | | | 1 |
| Learned from failures | 1 | | | | 1 |
| Helpful in understanding purpose of life | 1 | | | | 1 |
| | | | | ***Subtotal:*** | ***12*** |
| **Confidence:** | | | | | |
| Enhanced self-confidence | 6 | | | | 6 |
| Helpful for future career | 2 | | | | 2 |
| Enhanced self-efficacy | 1 | | | | 1 |
| Enhanced stress management | 3 | | | | 3 |
| Learned goal-setting and realization of goals | 1 | | | | 1 |
| | | | | ***Subtotal:*** | ***13*** |
| **Connection:** | | | | | |
| Improved relationship with peers, make more friends | 17 | | | | 17 |
| Improved communication skills and interpersonal relationships | 10 | | | | 10 |
| Improved communication and relationship with family | 7 | | | | 7 |
| Enhanced teacher-student relationship and understanding | 6 | | | | 6 |
| Enhanced feeling of being supported | 1 | | | | 1 |
| | | | | ***Subtotal:*** | ***41*** |
| **Competence:** | | | | | |
| Enhanced problem solving skills | 13 | | | | 13 |
| Enhanced ability in handling emotions | 9 | | | | 9 |
| Enhanced anger management, less impulsive | 4 | | | | 4 |
| Learned to do appropriate things at the right place | 3 | | | | 3 |
| Provided opportunities to share and express oneself | 4 | | | | 4 |
| Acquired refusal skills | 3 | | | | 3 |
| Could differentiate good friends from bad friends | 1 | | | | 1 |
| Learned how to treat people and deal with issues | 2 | | | | 2 |
| Learned positive thinking | 1 | | | | 1 |
| Learned critical thinking | 1 | | | | 1 |
| Increased awareness of public morals | 1 | | | | 1 |
| | | | | ***Subtotal:*** | ***42*** |

**TABLE 4 (continued)**

| | | | | | |
|---|---|---|---|---|---|
| **Compassion:** | | | | | |
| Enhanced respect for others | 1 | | | | 1 |
| Enhanced empathy | 1 | | | | 1 |
| | | | | ***Subtotal:*** | ***2*** |
| **Caring:** | | | | | |
| Used learned materials to help or teach others | 2 | | | | 2 |
| Less conflict and quarrel | 3 | | | | 3 |
| Enhanced sense of belongingness towards the school | 1 | | | | 1 |
| Learned teamwork | 1 | | | | 1 |
| Reduced bullying behavior | 2 | | | | 2 |
| | | | | ***Subtotal:*** | ***9*** |
| **Contribution:** | | | | | |
| Learned voluntary work | 1 | | | | 1 |
| Enhanced understanding of mother country | 2 | | | | 2 |
| | | | | ***Subtotal:*** | ***3*** |
| **Miscellaneous:** | | | | | |
| Learned something, learned many things | 2 | | | | 2 |
| | | | | ***Subtotal:*** | ***2*** |
| **Total Count (Percentage):** | **124 (96.1%)** | **1 (0.8%)** | **4 (3.1%)** | **0 (0%)** | **129 (100%)** |

Shek et al.[2] suggested that it is important to consider alternative explanations in the interpretations of qualitative evaluation findings (Principle 10). There are several possible alternative explanations for the present findings. First, the findings can be explained in terms of demand characteristics. However, this explanation is not likely because the informants were encouraged to voice their views without restriction and negative voices were in fact heard. In addition, as the teachers were not present, there was no need for the students to narrate in a socially desirable manner. The second alternative explanation is that the findings were due to selection bias. However, this argument is not strong as the schools and students were randomly selected. The third alternative explanation is that the positive findings were due to ideological biases of the researchers. As several safeguards were used to reduce biases in the data collection and analysis processes, this possibility is not high. Finally, it may be argued that the perceived benefits were due to other youth enhancement programs. However, this argument can be partially dismissed as none of the schools in this study participated in the major youth enhancement programs in Hong Kong, including the Adolescent Health Project and the Understanding the Adolescent Project. In addition, participants in the focus group interviews were specifically asked about the program effects of the P.A.T.H.S. Project only.

With reference to the argument of Shek et al.[2] that the authors should discuss the limitations of the qualitative evaluation studies conducted (Principle 12), the limitations of the study are outlined below. First, although the number of schools and students participating in the study can be regarded as acceptable using standards in the mainstream qualitative evaluation studies, it would be helpful if more schools and participants could be recruited. Second, in addition to the one-shot focus group interviews, it would be illuminating if regular and ongoing qualitative evaluation data could be collected. Third, although observation data have been collected[10], the inclusion of other qualitative evaluation strategies such as in-depth individual interviews would be helpful to further understand the subjective experiences of the program participants. Finally, although 11 principles proposed by Shek et al.[2] were upheld in this study, peer checking and member checking (Principle 8) were not carried out in this study because of time and manpower constraints. Despite these limitations, this study provides pioneering qualitative evaluation findings supporting the positive nature of the Project P.A.T.H.S. and its effectiveness in promoting holistic youth development among Chinese adolescents in Hong Kong.

## ACKNOWLEDGMENTS

The preparation for this paper and the Project P.A.T.H.S. were financially supported by the Hong Kong Jockey Club Charities Trust.

## References

1. Patton, M.Q. (1997) *Utilization-Focused Evaluation: The New Century Text.* Sage, Thousand Oaks, CA.
2. Shek, D.T.L., Tang, V., and Han, X.Y. (2005) Quality of qualitative evaluation studies in the social work literature: evidence that constitutes a wakeup call. *Res. Soc. Work Pract.* **15**, 180–194.
3. Shek, D.T.L. (2006) Adolescent developmental issues in Hong Kong: relevance to positive youth development programs in Hong Kong. *Int. J. Adolesc. Med. Health* **18(3),** 341–354.
4. Catalano, R.F., Berglund, M.L., Ryan, J.A.M., Lonczak, H.S., and Hawkins, J.D. (2002) Positive youth development in the United States: research findings on evaluations of positive youth development programs. *Prev Treatment* [serial on the Internet]. Jun 24 [cited 2004 Nov 17];5(15):[about 111 p.]. Available from: http://www.journals.apa.org/prevention/volume5/pre0050015a.html
5. Shek, D.T.L. Effectiveness of the Tier 1 Program of the Project P.A.T.H.S.: preliminary objective and subjective outcome evaluation findings. In *Holistic Adolescent Development: Development of a Positive Youth Development Program in Hong Kong*. Shek, D.T.L. and Merrick, J., Eds. Freund Publishing House, London, in press.
6. Denzin, K. and Lincoln, Y.S. (2005) *The Sage Handbook of Qualitative Research*. Sage, Thousand Oaks, CA.
7. Shek, D.T.L., Lam, M.C., Lam, C.M., and Tang, V. (2004) Perceptions of present, ideal, and future lives among Chinese adolescents experiencing economic disadvantage. *Adolescence* **39**, 779–792.
8. Stufflebeam, D.L. (2000) The CIPP model for evaluation. In *Evaluation Models: Viewpoints on Educational and Human Services Evaluation.* Stufflebeam, D.L., Medaus, G.F., and Kellaghan, T., Eds. Kluwer Academic, Boston. pp. 279–318.
9. Shek, D.T.L., Ng, H.Y., Lam, C.W., Lam, O.B., and Yeung, K.C. (2003) *A Longitudinal Evaluation Study of a Pioneering Drug Prevention Program (Project Astro MIND) in Hong Kong*. The University of Hong Kong.
10. Shek, D.T.L., Ma, H.K., Lui, J., and Lung, W.M. Process evaluation of the Tier 1 Program of the Project P.A.T.H.S. In *Holistic Adolescent Development: Development of a Positive Youth Development Program in Hong Kong*. Shek, D.T.L. and Merrick, J., Eds. Freund Publishing House, London, in press.
